# Spatial correlation between in vivo imaging and immunohistochemical biomarkers: A methodological study

**DOI:** 10.1016/j.tranon.2024.102051

**Published:** 2024-07-16

**Authors:** Hilde J.G. Smits, Edwin Bennink, Lilian N. Ruiter, Gerben E. Breimer, Stefan M. Willems, Jan W. Dankbaar, Marielle E.P. Philippens

**Affiliations:** a Department of Radiotherapy, University Medical Center Utrecht, Utrecht, the Netherlands; b Department of Radiology, University Medical Center Utrecht, Utrecht, the Netherlands; c Department of Pathology, University Medical Center Utrecht, Utrecht, the Netherlands; d Department of Pathology and Medical Biology, University Medical Center Groningen, Groningen, the Netherlands

**Keywords:** Dynamic contrast enhanced CT, Immunohistochemistry, Head and Neck Cancer

## Abstract

In this study, we present a method that enables voxel-by-voxel comparison of in vivo imaging to immunohistochemistry (IHC) biomarkers. As a proof of concept, we investigated the spatial correlation between dynamic contrast enhanced (DCE-)CT parameters and IHC biomarkers Ki-67 (proliferation), HIF-1α (hypoxia), and CD45 (immune cells). 54 whole-mount tumor slices of 15 laryngeal and hypopharyngeal carcinomas were immunohistochemically stained and digitized. Heatmaps of biomarker positivity were created and registered to DCE-CT parameter maps. The adiabatic approximation to the tissue homogeneity model was used to fit the following DCE parameters: $K^{trans}$ (transfer constant), $V_{e}$ (extravascular and extracellular space), and $V_{i}$ (intravascular space). Both IHC and DCE maps were downsampled to 4 × 4 × 3 mm[3] voxels. The mean values per tumor were used to calculate the between-subject correlations between parameters. For the within-subject (spatial) correlation, values of all voxels within a tumor were compared using the repeated measures correlation ($r_{rm}$). No between-subject correlations were found between IHC biomarkers and DCE parameters, whereas we found multiple significant within-subject correlations: $V_{e}$ and Ki-67 ($r_{rm}$ = -0.17, *P* < .001), $V_{e}$ and HIF-1α ($r_{rm}$ = -0.12, *P* < .001), $K^{trans}$ and CD45 ($r_{rm}$ = 0.13, *P* < .001), $V_{i}$ and CD45 ($r_{rm}$ = 0.16, *P* < .001), and $V_{i}$ and Ki-67 ($r_{rm}$ = 0.08, *P* = .003). The strongest correlation was found between IHC biomarkers Ki-67 and HIF-1α ($r_{rm}$ = 0.35, *P* < .001). This study shows the technical feasibility of determining the 3 dimensional spatial correlation between histopathological biomarker heatmaps and in vivo imaging. It also shows that between-subject correlations do not reflect within-subject correlations of parameters.

## Highlights

• We present a unique method of correlating in vivo imaging to immunohistochemistry.
• 3D heatmaps of biomarker presence are created from whole-mount tumor resections.
• By registering the 3D heatmaps to imaging, we can spatially compare them.
• The method provides insight into how well imaging portrays the tumor microenvironment.

## Introduction

Tumors are more than solely a proliferating mass of tumor cells. They are complex tissues consisting of multiple different cell types, interacting with each other in an atypical manner, forming the tumor microenvironment (TME) [1]. In head and neck squamous cell carcinoma, the TME consists of cancer stem cells, different types of immune cells, stromal cells, and endothelial cells that create an ecosystem for tumor cells to grow [[1], [2], [3]].

Immunohistochemistry (IHC) can be used as a readout for the biological processes occurring in the TME. Information about these processes can help diagnose and classify tumors, predict patient prognosis, and inform treatment decisions. However, in a pretreatment situation, IHC can only be performed on biopsy material. By definition, this only samples the tumor in one location and fails to account for intratumor heterogeneity often present in head and neck cancers [[4], [5], [6], [7], [8]].

Functional imaging is a collection of techniques that reflect physiological processes, like blood flow or metabolism, in vivo. These techniques can give us insight into the TME throughout the entire tumor volume, but the resolution of in vivo imaging is of course much lower than pathological assessment.

To determine how well radiological imaging reflects the TME, we set out to create a method that can correlate IHC biomarkers with in vivo imaging on a voxel-by-voxel basis. This can help validate imaging biomarkers. In this study, we compared the distribution of IHC biomarkers to dynamic contrast enhanced (DCE) computed tomography (CT). In DCE imaging, a series of images is acquired after intravenous injection of a contrast agent. The change in contrast over time measured in the tumor tissue is used to estimate properties of the tumor micro-vasculature, like intravascular space ($V_{i}$), extravascular and extracellular space ($V_{e}$), or the transfer constant ($K^{trans}$).

While multiple studies have compared DCE imaging variables with histological biomarkers in head and neck cancer [[9], [10], [11], [12], [13]], these studies use mean values of biomarker presence on biopsies to characterize tumors. This does not take into account intratumor heterogeneity. In our study, whole-mount tumor resections were used to create heatmaps of biomarker positivity that can be stacked and registered to imaging data. These stacked heatmaps facilitate a voxel-by-voxel comparison of parameters, allowing us to look at within-subject correlations. To characterize the tumor and the TME, three IHC biomarkers were used: Ki-67 (proliferation marker), HIF-1α (hypoxia marker), and CD45 (immune cell marker).

This article describes a novel method of spatially correlating in vivo imaging parameters with IHC parameters. To demonstrate proof of concept, we tested the ability of DCE-CT to reflect the TME of laryngeal and hypopharyngeal tumors. We do this by comparing DCE-CT imaging to whole-mount tumor resections and determining the within-subject (spatial) correlation between DCE-CT parameters and IHC biomarkers.

## Methods

### Patient selection

The patients included in this study were part of a previous study by Caldas-Magalhaes et al. [[14], [15], [16]]. Thirty-six patients with primary laryngeal or hypopharyngeal squamous cell carcinoma who were scheduled for a total laryngectomy (TLE) were included in the University Medical Center Utrecht between March 2008 and August 2014. The study was approved by the institutional review board.

### DCE-CT imaging

Before TLE, all patients underwent a DCE-CT in an immobilizing radiotherapy mask (Posicast PR5, Civco, Reeuwijk, The Netherlands). The scans were acquired on a Philips Brilliance iCT scanner (Philips Healthcare, Best, The Netherlands).

At the start of the scan, patients were injected with 50 mL of iodine contrast into the cubital vein at a rate of 5 mL/s, followed by a 40 mL saline flush at a rate of 5 mL/s. The DCE-CT was acquired with 120 kVp and 80 mAs per frame. First, 20 images were acquired at a three second interval, followed by 10 images at a six second interval and 10 images at a 20 second interval. The field-of-view covered the entire neck in the transverse plane, with a craniocaudal coverage of the entire tumor, ranging between 55 and 70 mm. The scans were reconstructed with a voxel spacing of 0.35 × 0.35 × 0.63 mm³.

Even though global movement was restricted by the radiotherapy mask, respiratory motion and swallowing still caused local movement of anatomical structures. To correct for this motion, elastic registration was performed after reconstruction using a groupwise b-spline registration in the Elastix toolbox [17,18]. The registered scans were smoothed using a guided filter with three guides [19].

The arterial input function was manually selected from the internal carotid artery and shows the influx of contrast agent over time in the artery. This function was used as input for the perfusion model as it represents the contrast arriving in the tumor.

In a perfusion model, perfusion behavior in tissues is modelled based on the arterial input function and the measured output (the contrast over time within the tumor). A previous study, performed on a partially overlapping patient pool, compared five perfusion models for estimating the transfer constant ($K^{trans}$) in larynx and hypopharynx tumors using DCE-CT [20].

This study found the Adiabatic Approximation to the Tissue Homogeneity model [21] to be the preferred model for DCE-CT parameter estimation. Because the individual contribution of intravascular space ($V_{i}$), and extravascular, extracellular space ($V_{e}$) to the DCE-CT signal is hard to separate in the tumor tissue, the mean intravascular transit time was fixed to four seconds.

In this model, parameter maps are generated for $K^{trans}$, $V_{i}$, and $V_{e}$, Supplementary Material 1, as well as the time-to-peak (TTP), the time until the maximum intravascular contrast is reached in a voxel.

### Surgical specimen and pathological procedures

After TLE, the surgical specimen was processed as previously described [14]. A complete overview of the pathological workup can be found in Supplementary Material 1. In short, the laryngectomy specimen was fixated in 4% formaldehyde for at least 36 h. After fixation, an ex vivo CT scan was made of the specimen on a Big Bore CT system (Philips Healthcare, Best, The Netherlands) before being embedded in an agar block. This block was sliced into 3 mm tissue blocks in the axial plane on a conventional meat slicer. Each 3 mm slice was photographed.

### Immunohistochemistry

For each specimen, the four consecutive 3 mm slices that contained the largest tumor diameter were chosen for analysis. From these slices, 4 µm sections were cut for whole-mount hematoxylin and eosin (H&E) staining, as well as for 3,3′-diaminobenzidine (DAB) IHC staining for the biomarkers Ki-67, CD45 and HIF-1α. The IHC staining was performed as previously described [22,23]. For the Ki-67 staining, Mouse anti-Ki67 monoclonal antibody was used (DAKO, Agilent (Santa Clara, California, USA), 1:100 dilution, 60 min incubation, Clone: MIB-1 20,027,876). For the CD45 staining, Mouse anti-Human CD45 monoclonal antibody was used (DAKO, Agilent 1:200 dilution, 60 min incubation, Clone: PD7/26+2B11). For the HIF-1α IHC, the Novolink kit (Leica Biosystems, Rijswijk, the Netherlands) was used with the primary antibody Mouse- anti-HIF-1α (BD Biosciences (Franklin Lakes, New Jersey, United States of America), 1:50 dilution, overnight incubation, Clone: cat# 610,959, lot 4 073 775).

### Immunohistochemistry image processing

After IHC staining, all whole-mount tissue sections were digitized using the Aperio ScanScope XT scanner (Leica Biosystems, Wetzlar, Germany) at 40x magnification. Image processing of the IHC sections was performed in QuPath version 0.3.0 [24].

**Fig. 1:**
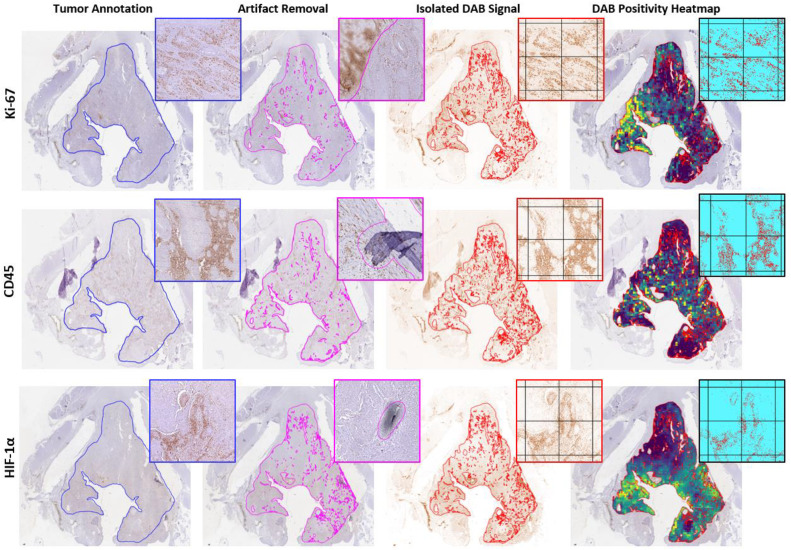
Image processing of IHC stained tumor sections to heatmaps of biomarker positivity. The tumor is delineated on the Ki-67 section and transferred to the registered CD45 and HIF-1α section (blue). Staining and scanning artifacts are manually removed on all sections, which creates three tumor annotations without artifacts on each section (pink). The intersection of the three corrected annotations (red) is used for analysis of the isolated DAB signal. Tiles of 0.5 × 0.5 mm^2^ are created. The percentage of positive pixels (pixels with an optical density of the DAB signal above the threshold of 0.18 for Ki-67, 0.21 for CD45, or 0.20 for HIF-1α) in each tile is used to create a heatmap of biomarker positivity.

Tumors were manually delineated on the Ki-67 section under the supervision of a dedicated head and neck pathologist (G.E.B.). The CD45 and HIF-1α sections were rigidly registered to the corresponding Ki-67 section of each tumor slice. Artifacts like tissue fold-over, nonspecific staining, and scanning artifacts on any of the IHC sections were removed from the annotation. The final tumor annotation of each slice only contained areas without artifacts on any of the matched IHC stained sections Fig. 1.

The tumor annotation was divided into 0.5 × 0.5 mm^2^ tiles. Tiles located at the border of the tumor were cropped to fit the contour of the tumor annotation. Tiles with a usable area of less than 50% after cropping were removed from analysis, Supplementary Material 1.

To negate the effect of inter-batch differences in staining intensity, a threshold was used to determine the positivity of each tile Fig. 1. First, the DAB color signal was isolated using a color deconvolution method [25]. For each biomarker, a composite image was created of tiles from different tumors, Supplementary Material 1. These tiles were chosen to include areas with high and low biomarker presence and high and low DAB intensity. By visual inspection of these composite images, a dedicated head and neck pathologist (S.M.W.) determined the optimal threshold of DAB optical density to indicate positive pixels. This threshold was 0.18 for Ki-67, 0.21 for CD45, and 0.20 for HIF-1α staining. The tiles of the IHC slices were converted into heatmaps of DAB positivity.

### Image registration

**Fig. 2:**
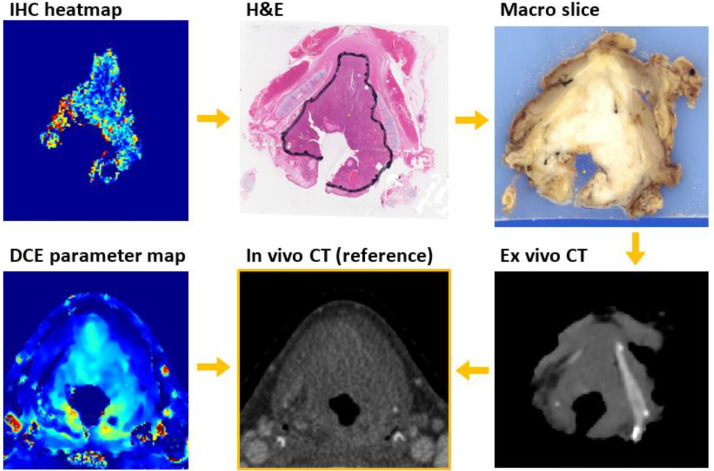
Registration procedure of IHC slices to the in vivo imaging. The in vivo CT is used as the reference image.

A complete overview of the image registration can be found in Supplementary Material 1. Each IHC heatmap was manually registered to its H&E counterpart Fig. 2. Registration of the H&E sections to the in vivo imaging has been performed and validated previously [14,15]. In short, each H&E section was manually registered to the corresponding photographed macroscopic slice. The macroscopic slices were stacked to create a 3D digital reconstruction of the specimen. This reconstruction was registered to the ex vivo CT scan of the specimen using the Elastix toolbox [17,18]. The ex vivo CT was registered to the in vivo CT using the outlined thyroid and cricoid cartilage as a reference. As the rigid cartilage helped maintain the shape and size of the enclosed tissues, large deformations were only found in tumors with larger distances from the cartilage structure [14]. If such deformations were present in the four slices used for IHC analysis, the slices were excluded from analysis.

The registered IHC heatmaps of individual tumor slices were stacked to form 3D heatmaps. As the thickness of the macroscopic tumor slices was 3 mm, we used this as the slice thickness of the 3D heatmaps, resulting in a voxel size of 0.5 × 0.5 × 3 mm^3^. The 3D IHC heatmaps and the DCE-CT parameter maps were resampled to the grid of the in vivo CT scan (reference image), so each voxel represents the same anatomy.

## Analysis

The IHC heatmaps and the DCE-CT parameter maps were exported into MATLAB R2019a (MathWorks, Natick, MA) and further processed with in-house developed software. Only the overlapping volume was used in the analysis, representing the tumor volume as delineated on the IHC sections.

Laryngeal and hypopharyngeal tumors are likely to border cartilage or air cavities. Since DCE parameters cannot be accurately determined in these areas, the parameter maps are likely to contain artifacts around the edges of the tumor due to residual motion artifacts. Voxels within the tumor area with TTP, $V_{i}$, $V_{e}$ or $K^{trans}$ values that were zero or unusually high were removed from analysis, as these are likely artifacts. To determine the upper limit of each DCE parameter, histograms were made of the voxels values from all tumors grouped together. The cut-off value of each parameter was the value two standard deviations (σ) removed from the mean (μ). Additionally, $V_{i}$ and $V_{e}$ were cut off at a physiological limit of 100 mL/100 g if μ+2σ exceeded this threshold.

A previous study that validated the registration method found an in-plane root mean square error (RMSE) of 1.94 mm^2^ when registering H&E sections to in vivo CT [14]. As the RMSE corresponds to the standard deviation of errors, two times the RMSE covers about 95% of registration errors. Therefore, the in-plane resolution was downsampled to create 4 × 4 × 3 mm^3^ voxels. If a downsampled voxel only contained 10 or less of the original 0.5 × 0.5 × 3 mm^3^ voxels, the voxel was excluded to avoid edge artifacts, Supplementary Material 1.

### Statistical analysis

Since the mean transit time was fixed in the model we used, the TTP only reflects the arterial bolus delay and is no longer tissue-specific. For this reason, TTP was left out of the analysis.

All correlations between the IHC parameters (Ki-67, HIF-1α, and CD45) and the remaining DCE-CT parameters ($K^{trans}$, $V_{i}$ and $V_{e}$) (*n* = 9 correlations) were included in the analysis. Additionally, we looked at the correlations between the three IHC parameters (*n* = 3 correlations).

Two types of correlations were investigated: between-subject and within-subject correlations. The between-subject correlation between parameter pairs was computed using the subject means in MATLAB R2019a. Pearson's correlation coefficient r was calculated, and student *t*-tests were used to test whether the null hypothesis (r = 0) held. To correct for multiple testing, a Bonferroni correction was applied to the significance level. Since we performed 12 tests, $P<\frac{.05}{12}=0.004$ was deemed a significant correlation.

The within-subject correlations between the parameters were determined with the repeated measures correlation $r_{rm}$ using the rmcorr package in R [26,27]. Each voxel is a repeated measure of all DCE-CT and IHC parameters. The rmcorr method determines the within-subject association between paired repeated measures for multiple subjects [27]. With the Bonferroni correction, P<0.004 was deemed a significant result.

## Results

A total of 36 patients participated in the original study [16], of which 15 were included in the final analysis of the current study. Reasons for exclusion were: patients did not undergo pretreatment DCE-CT (7), patients were part of the protocol optimization test set (6), severe movement during the DCE-CT imaging (2), insufficient registration accuracy between pathology and imaging (2), significant tumor debulking was done between in vivo imaging and TLE (1), the tumor was too large for the whole-mount procedure (1), the tumor fragmented during surgery (1), or there was inadequate DAB staining throughout the entire tumor (1).

The included patients were all male with a median age of 61 years (range: 51–79). Eight larynx and seven hypopharynx tumors were included, 14 with a T4 tumor stage and one with a T3 tumor stage. The median time between DCE-CT and surgery was one day (range: 1–34 days).

Of the 60 available whole-mount slices, 54 were included in the analysis, with a minimum of two tumor slices per patient. Reasons for exclusion were out of focus scans (3), too many tissue artifacts (1), inadequate DAB staining (1), or fragmented tissue on the slice (1).

The cut-off points for DCE-CT parameters were 18.51 s for TTP, 14.34 mL/100 g for $V_{i}$, and 100 mL/100 g for $V_{e}$, Supplementary Material 2. Since the $K^{trans}$ parameter maps did not contain outliers, no cut-off point was used for $K^{trans}$. Tiles with values that exceeded any of these cut-offs were removed from analysis. The median number of tiles included in the analysis was 78 per tumor (range: 31–213). The distribution of the DCE and IHC parameters throughout our cohort is shown in Table 1.

**Table 1:** Median DCE and IHC tumor parameters of all patients.

DCE parameters		Median (range)
$K^{trans}$	[ml/100 g/min]	18.4 (11.2 – 30.8)
$V_{e}$	[ml/100 g]	30.3 (18.5 – 41.5)
$V_{i}$	[ml/100 g]	5.1 (2.2 – 7.3)

IHC parameters		
Ki-67	[% positive pixels]	5.1 (1.9 – 11.3)
HIF-1α	[% positive pixels]	2.2 (0.7 – 7.7)
CD45	[% positive pixels]	3.7 (0.2 – 16.7)

### Between-subject correlation

**Fig. 3:**
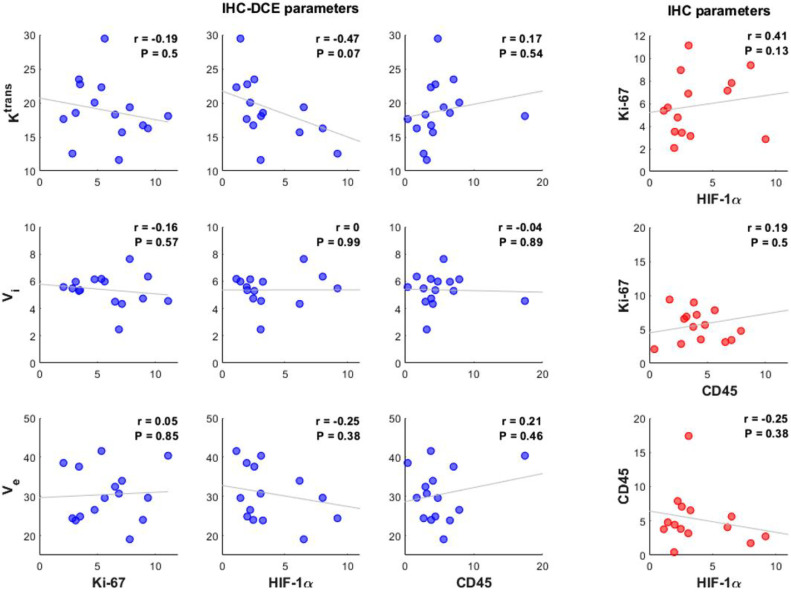
Correlations between mean IHC biomarker positivity in percentage of positive pixels and mean DCE values per patient (*n* = 15) in blue. Correlations between mean IHC biomarker positivity per patient in red. The Pearson's correlation coefficient r and the significance P is given in the top right corner of each graph. No significant correlations were found. $K^{\text{trans}}$: transfer constant (mL/100 g/min), $V_{i}$: intravascular space (mL/100 g), $V_{e}$: extravascular and extracellular space (mL/100 g).

When looking at the mean values of the IHC positivity and DCE parameters per tumor, no significant between-subject correlations were found Fig. 3.

### Within-subject correlation

**Fig. 4:**
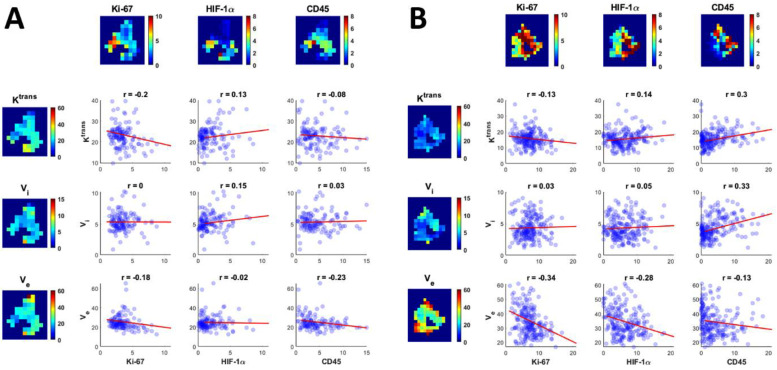
Examples of the spatial correlations between IHC biomarker expression (in percentage of positive pixels) and DCE parameters in two patients. The heatmaps show the parameter distribution in one tumor slice of both patients, while the scatterplots show the correlation between all 4 × 4 × 3 mm^3^ voxels from each patient (*n* = 130 voxels for patient A and *n* = 210 voxels for patient B). The least square regression line is shown in red and the Pearson's correlation coefficient r is added for each parameter pair.$\;K^{\text{trans}}$: transfer constant (mL/100 g/min), $V_{i}$: intravascular space (mL/100 g), $V_{e}$: extravascular and extracellular space (mL/100 g).

As for the within-subject correlation, Fig. 4 shows the correlations between IHC biomarkers and DCE parameters in two patients. In general, patient A has a higher $K^{trans}$ and a lower $V_{e}$ than patient B. Patient A also shows less Ki-67 and HIF-1α expression than patient B. When looking at the correlations, we see an overall agreement between the two patients when comparing, for example, $V_{e}$ and Ki-67 (Pearson's r = −0.18 for patient A and r = −0.34 for patient B). However, there are also some disparities, e.g. the correlation between $K^{trans}$ and CD45 (r = −0.08 in patient A and r = 0.3 in patient B). This may indicate inter-tumor heterogeneity in this patient group.

**Fig. 5:**
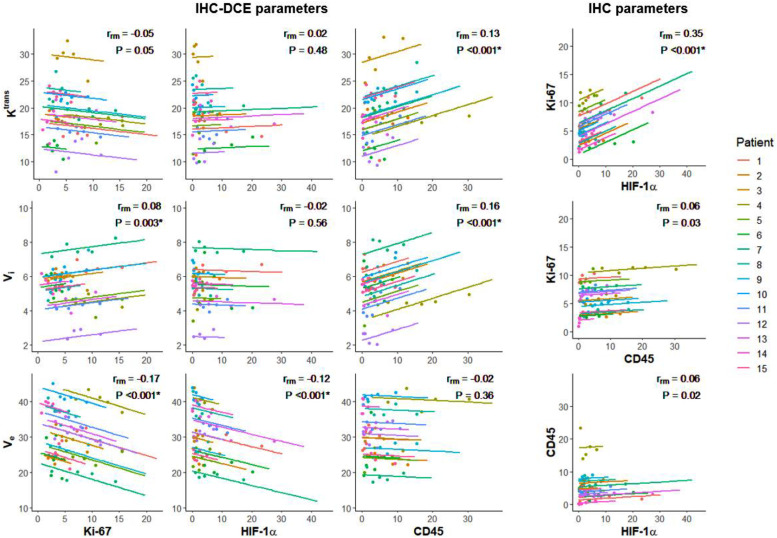
Repeated measures correlation plots for all parameter pairs. For each patient, data points were sorted into five equally sized bins whose means are shown in the graph, but the repeated measures correlation coefficient ($r_{\text{rm}}$) is based on all available voxels. *P* < .004 is deemed a significant result, indicated by *. The IHC parameters are represented in percentage of positive pixels, $K^{\text{trans}}$: transfer constant (mL/100 g/min), $V_{i}$: intravascular space (mL/100 g), $V_{e}$: extravascular and extracellular space (mL/100 g).

In order to find shared spatial correlations between all patients and to account for the inter-patient off-set differences, the repeated measures correlation coefficient was determined for all parameter pairs Fig. 5.

Multiple significant spatial correlations were found between IHC biomarkers and DCE parameters. Two negative spatial correlations: $V_{e}$ and Ki-67 ($r_{rm}$ = −0.17, *P* < .001) and $V_{e}$ and HIF-1α ($r_{rm}$ = −0.12, *P* < .001), and three positive spatial correlations: $K^{trans}$ and CD45 ($r_{rm}$ = 0.13, *P* < .001), $V_{i}$ and CD45 ($r_{rm}$ = 0.16, *P* < .001), and $V_{i}$ and Ki-67 ($r_{rm}$ = 0.08, *P* = .003), Fig. 5. Despite being all significant, the correlations were weak.

The strongest correlation found was between two IHC biomarkers: Ki-67 and HIF-1α ($r_{rm}$ = 0.35, *P* < .001).

## Discussion

This methodological study shows the technical feasibility of determining the within-subject spatial correlation between in vivo imaging and the ex vivo histopathological biomarker distribution. While previous studies correlated mean values or did histogram analyses, 3-dimensional voxel-by-voxel correlation of both in vivo and ex vivo biomarker maps is innovative. Full spatial correlation of in vivo imaging and histopathology can provide insight into various imaging techniques and their ability to portray the TME.

In the age of personalized medicine, more and more treatments become available based on the presence of specific biomarkers within a tumor. As treatment decisions are often based on the information of a single biopsy, intratumor heterogeneity challenges the efficacy of these treatments [8,28]. If further researched with appropriate biomarkers, techniques like the one presented in this paper could help identify whether a biopsy is representative of the entire tumor.

DCE imaging can play an important role in translational research, as it depicts physiological attributes of the tumor. It has been used for prediction of biomarker presence [12,13] and treatment outcome [29]. A recent randomized controlled trial also found positive treatment outcomes in a group that received radiotherapy boosts on tumor volumes with low blood volumes [30], underlying the clinical value of DCE imaging.

Multiple methods exists of correlating in vivo imaging to histopathology [31]. However these methods are almost exclusively used to validate tumor delineations [[32], [33], [34]]. While this is a relevant application, co-registered imaging and pathology data can also be used to validate imaging biomarkers. A method by Al-Mubarak et al. (2019) [35] stacked pathology sections in a way that facilitated voxel-by-voxel comparison to imaging parameters. This study achieved a comparison at an in-plane resolution of 113 × 113 µm^2^ in pre-clinical mouse models. The present study is the first to apply a voxel-by-voxel analysis in human patients. While the resolution of our analysis is lower due to motion artifacts and the scale of the resected material, we show the feasibility of such an analysis in humans.

In recent years, multiple studies have compared IHC biomarkers to DCE imaging in head and neck squamous cell carcinomas [[9], [10], [11], [12], [13]]. However, most studies evaluate the between-subject correlation by comparing a biomarker score from biopsy material to the mean imaging parameters within a tumor volume. This study is the first to determine the spatial, within-subject correlation between histopathological data and in-vivo DCE imaging in head and neck tumors.

One of our main findings is the discrepancy between the between-subject correlation of parameters and the within-subject correlations. No between-subject correlations were found between any of the parameter pairs evaluated, while multiple significant within-subject correlations were found. These findings indicate that an absence of a between-subject correlation between parameters does not mean these parameters are entirely independent. When reducing an entire tumor to one data point (e.g. mean expression of a certain biomarker), a lot of information about the complexity of the TME is lost. Methods like the one described in this paper are needed to capture the intra-individual patterns and take the intratumor heterogeneity and systematic errors into account.

The strongest within-subject correlation was found between the histopathological biomarkers Ki-67 and HIF-1α ($r_{rm}$ = 0.35). This is a somewhat surprising result; to our knowledge, this has not been described in literature before. HIF-1α is generally seen as a marker for acute hypoxia [36,37]. This type of hypoxia occurs when small blood vessels (temporarily) shut down, limiting blood supply to cells [36]. This is not a rare occurrence in tumors, as the tumor vasculature is often rapidly formed, resulting in abnormal, immature, and leaky vessels [38]. Highly proliferating cells causing an increased cell density might increase the chance of vessel occlusions, resulting in the observed spatial correlation between Ki-67 and HIF-1α. However, the activation of HIF-1α is a complex process, and further research is necessary before drawing any conclusions.

When looking at the within-subject correlations of IHC biomarkers with DCE-CT parameters, multiple significant correlations were found. The extravascular, extracellular space $V_{e}$ was negatively correlated with both Ki-67 and HIF-1α ($r_{rm}$ = −0.17 and $r_{rm}$ = −0.12, respectively). This is a fairly logical result, as it is to be expected that increased cell proliferation (and thus increased hypoxia) leads to less extracellular space in a tissue.

Significant spatial correlations were also found between CD45 and the DCE-CT parameters $K^{trans}$ and $V_{i}$ ($r_{rm}$ = 0.13 and $r_{rm}$ = 0.16, respectively). The presence of leukocytes is known to increase vascular permeability [39,40] and since immune cells are transported intravenously, a spatial correlation between CD45 and $V_{i}$ might thus be explained.

Additionally, we found a significant spatial correlation between $V_{i}$ and Ki-67 ($r_{rm}$ = 0.08), which might reflect increased angiogenesis in tumor regions with a lot of proliferation.

The fact that most correlations can be explained might indicate that in vivo DCE-CT gives relevant information about the tumor microenvironment. However, all correlations found between IHC biomarkers and DCE-CT parameters were quite weak, making it difficult to draw any conclusions regarding the feasibility of characterizing the TME with DCE-CT imaging. A larger patient population is needed to validate our results.

In the literature, we did not find any studies examining the spatial correlation between IHC and DCE parameters in head and neck carcinomas. However, in line with our results, two recent studies also found no between-subject correlations between Ki-67 positivity in biopsy material and mean $K^{trans}$ and $V_{e}$ values on DCE-MRI [9,11]. One of those studies also included HIF-1α and also found no between-subject correlation between HIF-1α and $K^{trans}$ or $V_{e}$ [11]. Histogram analysis of DCE parameters found a correlation between HIF-1α and the kurtosis of $K^{trans}$, but not the mean [12]. This further indicates that the spatial distribution of DCE parameters is relevant when comparing them to IHC biomarkers.

These studies use DCE-MRI data instead of DCE-CT imaging. While most studies agree that $K^{trans}$ is a robust parameter across different modalities, there are mixed results regarding the correlation of other DCE parameters when determined on MRI or CT [[41], [42], [43], [44]]. This might be attributed to the non-linear conversion of the signal intensity to contrast agent concentration in DCE-MRI, while DCE-CT has a direct read-out of contrast concentration. However, in clinical practice, DCE-MRI can be easily incorporated in any scanning protocol which uses gadolinium contrast agent. DCE-CT requires an extra scan that leads to more ionizing radiation exposure.

## Limitations

A considerable limitation of this method is that IHC biomarkers must be specific for a robust digital analysis. Initially, we also included CD31 as a vascular marker in this study since CD31 is expressed on endothelial cells. However, CD31 is also expressed on leukocytes [45]. Automatic detection of such non-specific markers on whole-mount tumor sections requires an additional step of separating staining of interest from unwanted biomarker staining. In previous studies where CD31 was used as a vascular marker, the staining was either assessed visually by pathologists or digitally on a preselected tumor region without artifacts or leukocytes [46,47]. Therefore, this method is less reliable when used to assess non-specific biomarkers.

Another limitation is the registration accuracy. In this proof of concept, we downsampled the data to compensate for registration errors. An improved registration method would allow for higher resolution voxels that can be compared. While deformations that occur during specimen resection or fixation are difficult to overcome, improved registration can be achieved by matching the pathological slices to the imaging slices using for example 3D-printed cutting molds [31,48].

When creating the IHC heatmaps, absolute DAB intensity thresholds are used to determine whether or not pixels are positive. These thresholds are vulnerable to intensity inhomogeneity, artifacts, noise, and inter-batch variability in staining intensity. Ideally, color normalization of the DAB staining should be performed before analysis [49]. However, in this analysis, we are mainly interested in relative intensity differences of DAB staining within a tumor, as we use repeated measures correlations, which are less affected by inter-batch staining variability.

As for the DCE-CT analysis, we chose a model with a fixed mean intravascular transit time because this parameter is difficult to estimate without a high temporal scanning resolution [50]. However, in reality, the transit time is a free model parameter. Because we fixed this parameter to four seconds, the effects of blood flow and the transit time are lumped into the intravascular blood volume parameter $V_{i}$ and information about their separate values is lost. Since tumors are heterogeneous in nature, it is reasonable to assume that the transit time varies within the tumor. Unfortunately, this heterogeneity cannot be determined using this CT acquisition protocol.

Additionally, since the original DCE-CT scans have a low signal-to-noise ratio, the imaging was filtered with an anisotropic guided filter [19]. In doing so, some of the spatial resolution of the in vivo scans was lost. The IHC sections were also downsampled before analysis, but this was only possible within the 4 µm sections and not through plane.

## Conclusions

This study shows the technical feasibility of correlating IHC biomarkers with in vivo imaging on a voxel-by-voxel basis. We also show that the between-subject correlation of mean parameters does not reflect the within-subject correlations of these parameters. Additionally, we show that DCE imaging might be able to portray TME characteristics, but a larger dataset is needed.

## Funding statement

The material used in this paper was collected in a study funded by the 10.13039/501100004622Dutch Cancer Society (KWF) Research Fund, project number 2011–5152. Additional funding for analysis was provided by the same society, project number 2017–10978.

## CRediT authorship contribution statement

**Hilde J.G. Smits:** Writing – review & editing, Writing – original draft, Visualization, Validation, Software, Methodology, Investigation, Formal analysis, Data curation, Conceptualization. **Edwin Bennink:** Writing – review & editing, Software, Methodology, Formal analysis. **Lilian N. Ruiter:** Writing – review & editing, Investigation, Data curation. **Gerben E. Breimer:** Writing – review & editing, Supervision. **Stefan M. Willems:** Writing – review & editing, Supervision, Resources, Conceptualization. **Jan W. Dankbaar:** Writing – review & editing, Supervision, Methodology. **Marielle E.P. Philippens:** Writing – review & editing, Supervision, Resources, Project administration, Methodology, Funding acquisition, Conceptualization.

## Declaration of competing interest

The authors declare that they have no known competing financial interests or personal relationships that could have appeared to influence the work reported in this paper.

## Appendix Supplementary materials

Image, application 1
