# Supplementary material for: Spatial correlation between in vivo imaging and immunohistochemical biomarkers: A methodological study
Source: Transl Oncol. 2024 Jul 16;48:102051. doi: 10.1016/j.tranon.2024.102051 (PMC11301398; doi:10.1016/j.tranon.2024.102051)
Supplement: Supplementary file 1 [file mmc1.docx]

Supplementary Material 1 - Methods

**Laryngectomy specimen preparation**

The laryngectomy specimen is collected from the operating room and fixated in 4% formaldehyde for at least 36 hours (Figure S1-1). After fixation, an ex vivo CT scan is made of the specimen, after which the specimen is embedded in agar. After the agar has hardened overnight, a corner of the agar block was cut off over the entire length block. This notch helps with aligning the slices when digitally reconstructing the specimen. The block is than sliced into 3 mm thick tissue blocks, the so-called macro slices. Each slice was measured with a digital caliper.


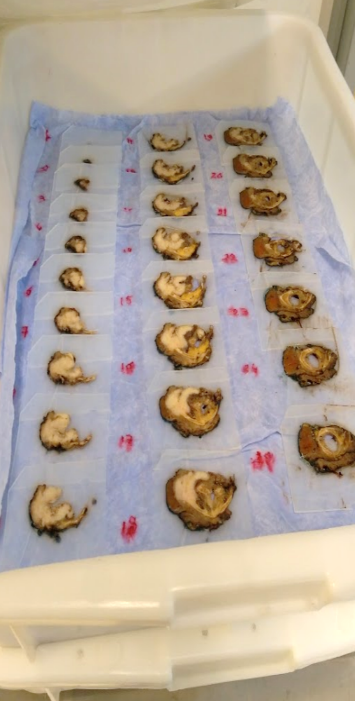

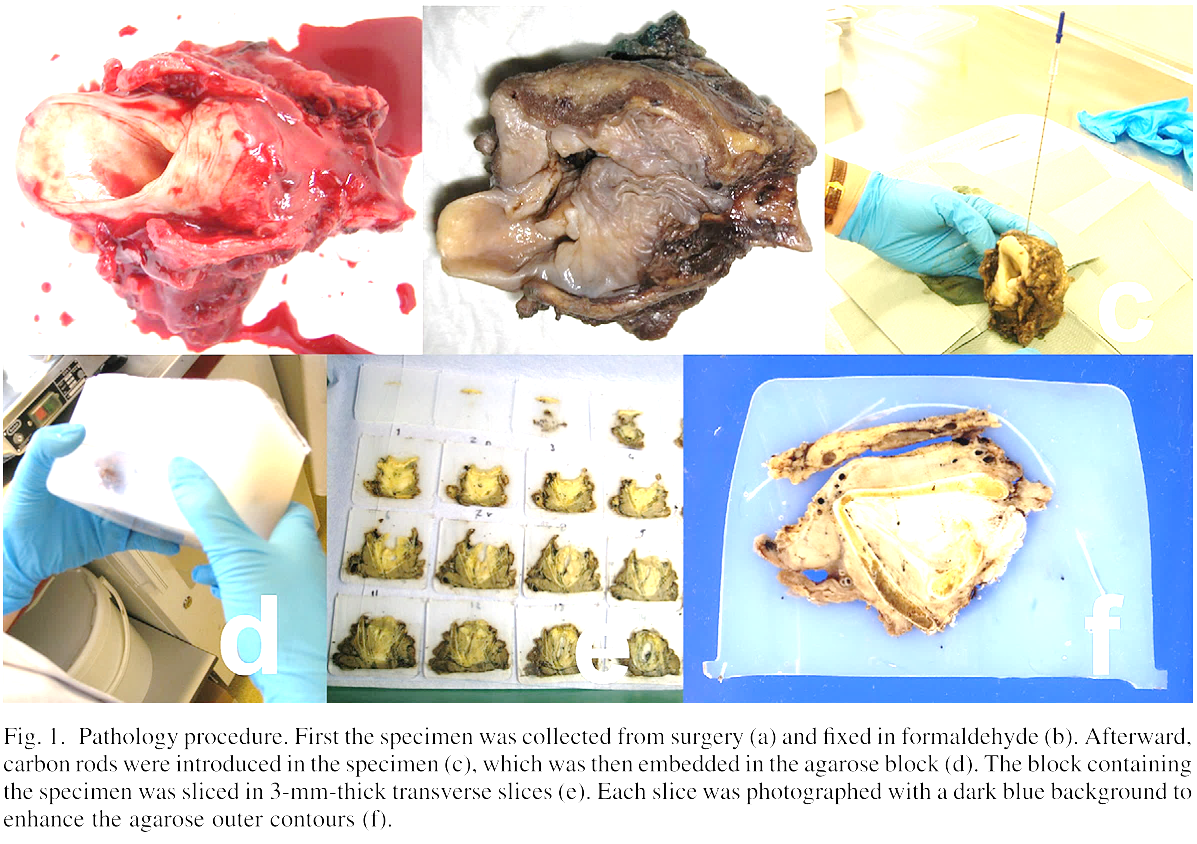

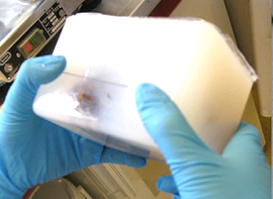

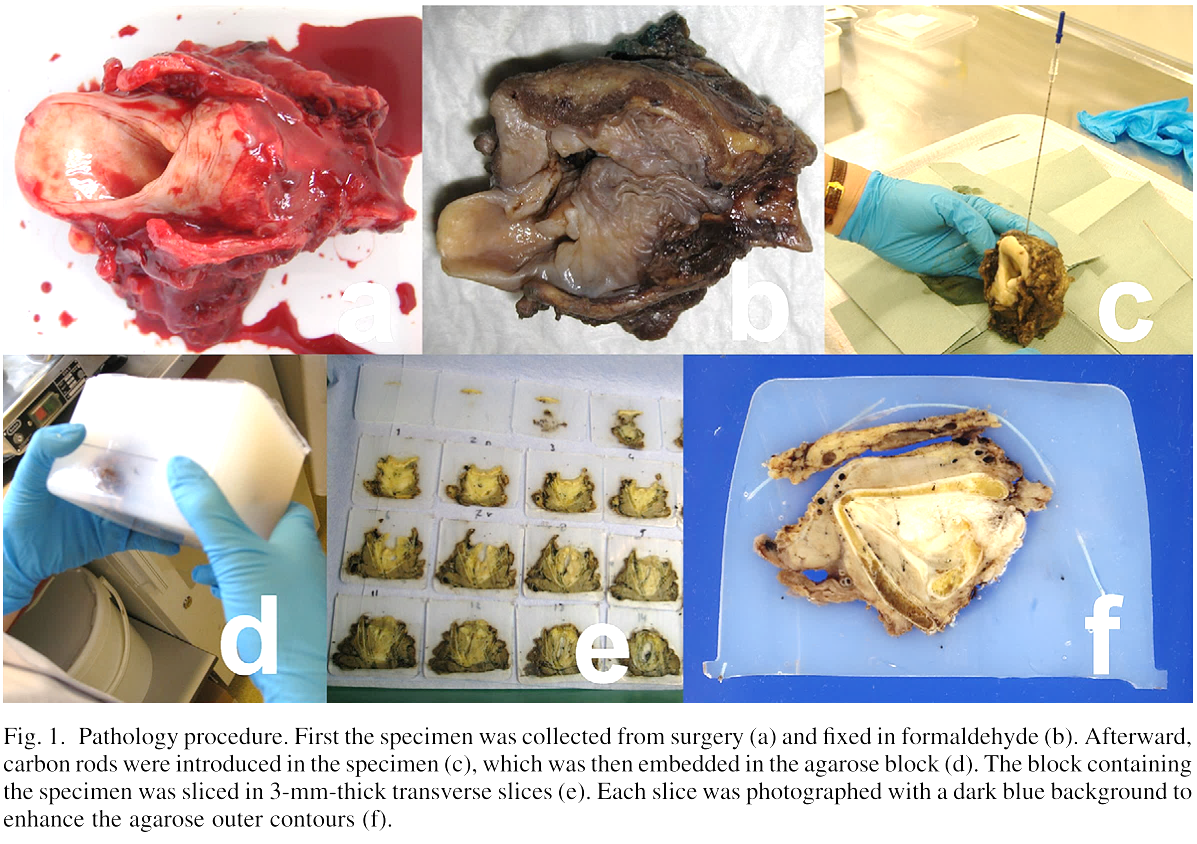


**A B C D**

*Figure S1-1: Preparation of laryngectomy specimen. The specimen (A) is collected and fixated in formaldehyde (B). The specimen is cast into an agar block (C), before being cut into 3mm think slices (D).*

**Digital reconstruction 3D specimen**

Each slice was photographed on a blue background to maximize the contrast between the edges of the agar slice and the background (Figure S1-2). For each laryngectomy specimen, one photograph included a ruler from which the resolution of the photograph could be derived.

The photographs were than stacked on top of each other by registering the agar edges of each slice to make sure the slices were aligned. The average slice thickness was used as the resolution in the Z-direction. Stacked together, the slices create a three dimensional reconstruction of the laryngectomy specimen, allowing for a coronal and sagittal view (Figure S1-2 D and E).


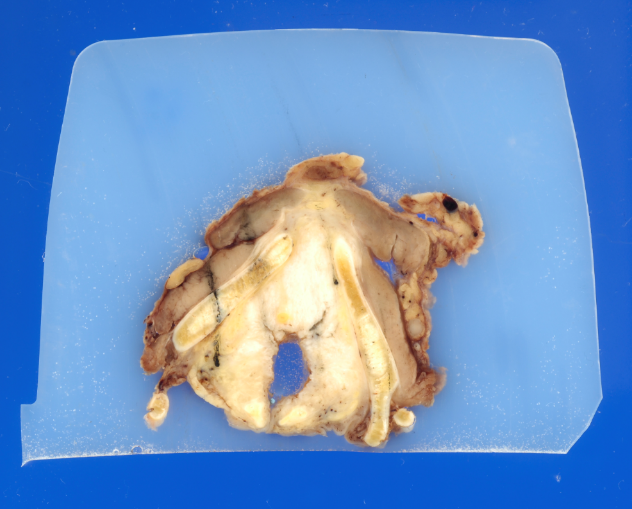

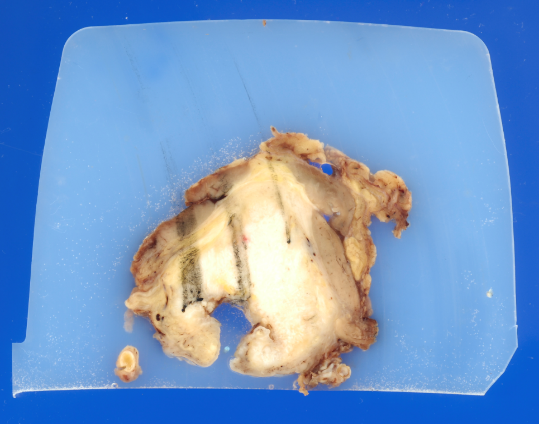

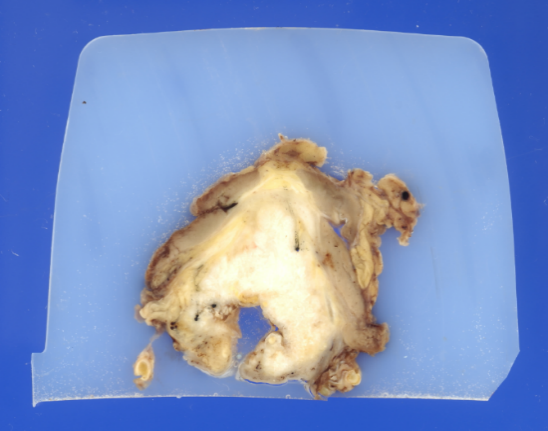

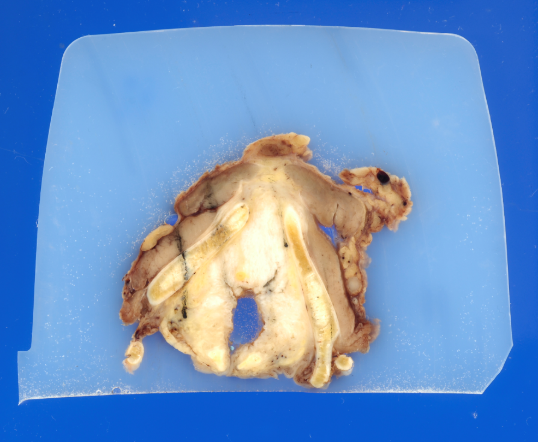

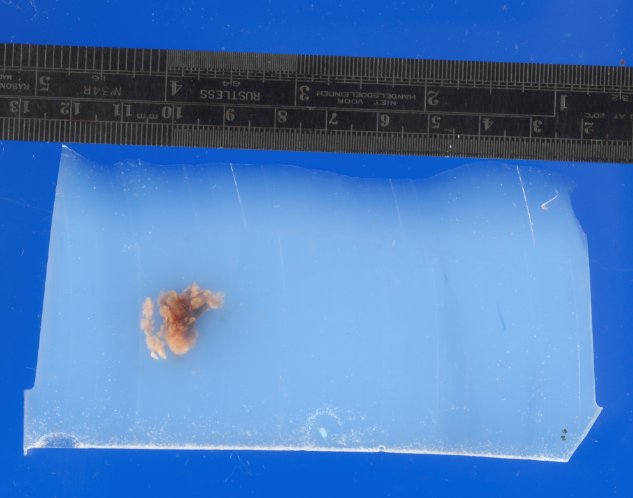

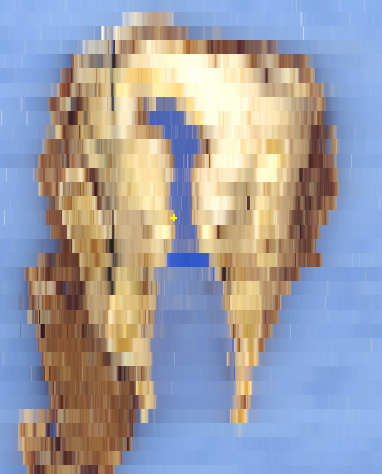

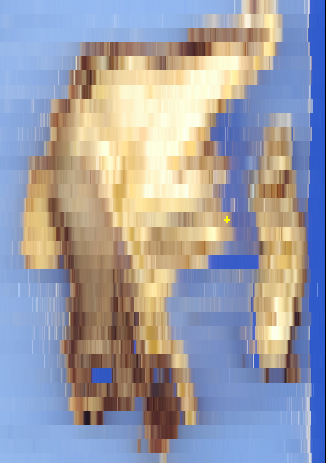


**A B**

**C D E**

*Figure S1-2: Digital reconstruction of the laryngectomy specimen. Photographs of the macro slices (A) are stacked to create a 3D reconstruction of the specimen (C), allowing for a coronal (D) and sagittal view (E). A ruler is included in the photographs to determine the resolution (B).*

**Histopathology**

From the macro slices, all slices containing tumor were selected and embedded in whole mount cases (green and orange in Figure S1-3). Additionally, the three slices above and below the slices with visible tumor (blue in Figure S1-3) were embedded as well to make sure the entire tumor and possible micro invasions were embedded. From these slices, 4 µm sections were cut and stained with hematoxylin and eosin (H&E).

From the center of the tumor, the four slices that contained the largest tumor diameter were chosen for immunohistochemical staining (green in Figure S1-3). From these slices, three additional 4 µm sections were cut and IHC stained for Ki-67 (proliferation marker), HIF-1α (hypoxia marker), and CD45 (immune cell marker) (Figure S1-4).

= Laryngectomy specimen

= Tumor

= Slice

= Slices with biggest tumor diameter

= Slices with visible tumor

= Three slices above and below visible tumor

*Figure S1-3: Schematic representation of tumor slices*

**Macro slice H&E Ki-67**

**CD45 HIF-1α**


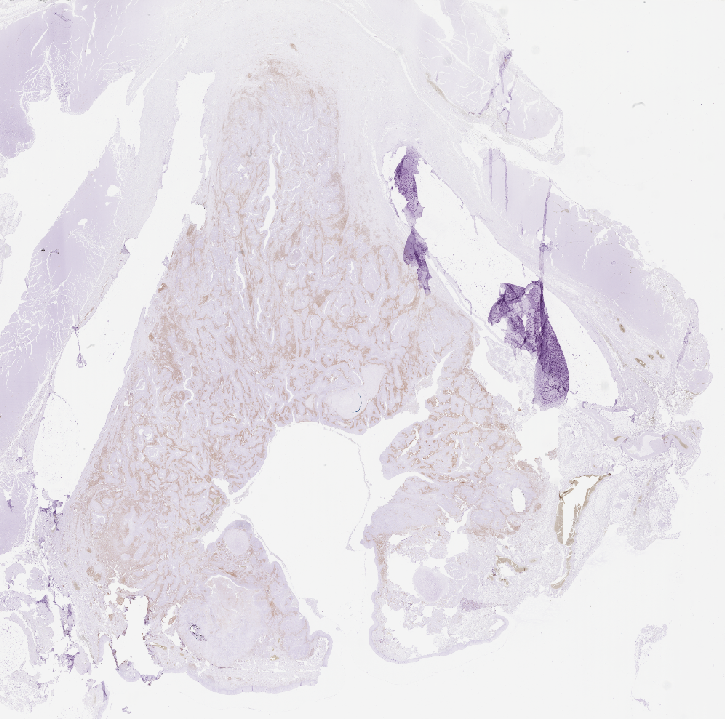

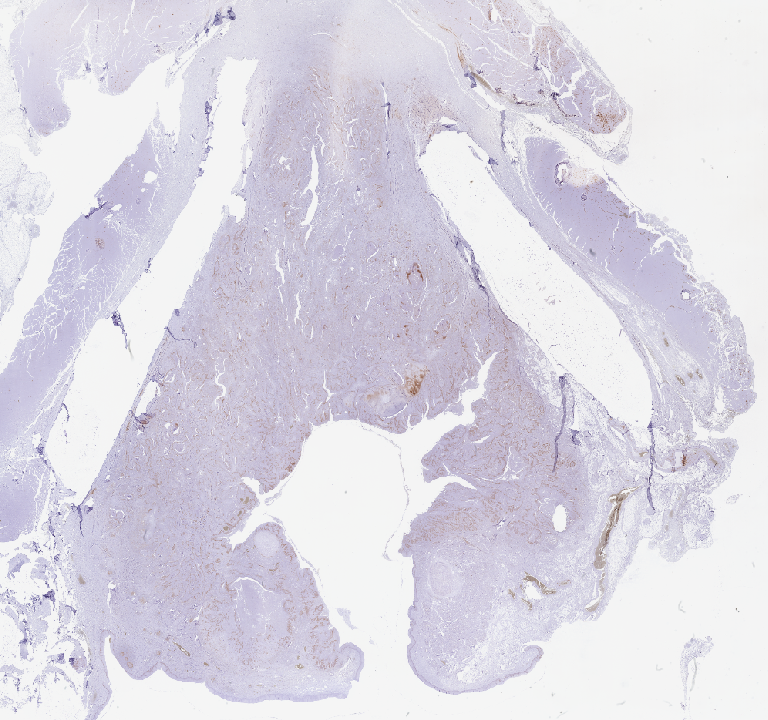

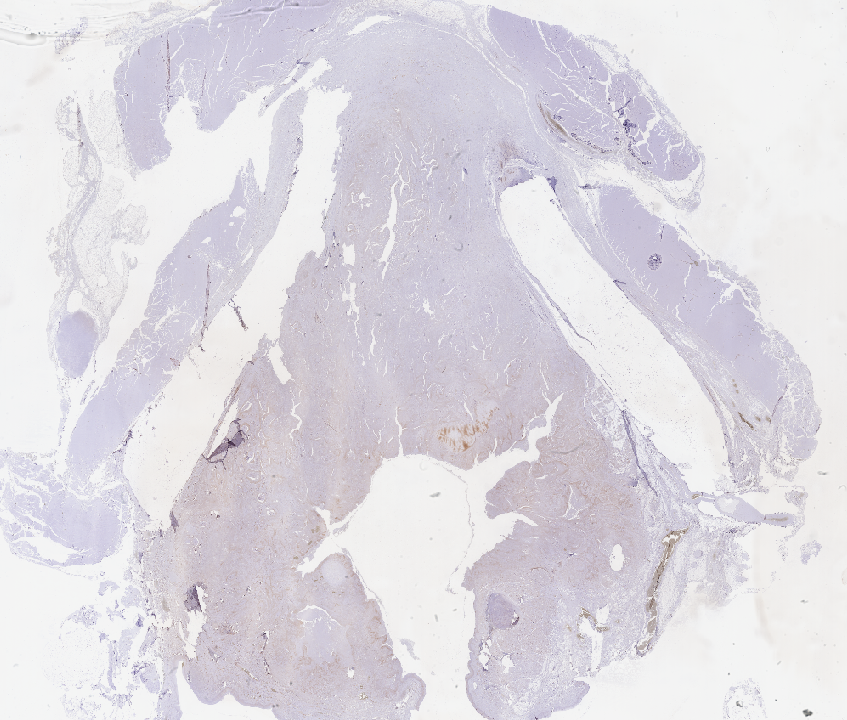

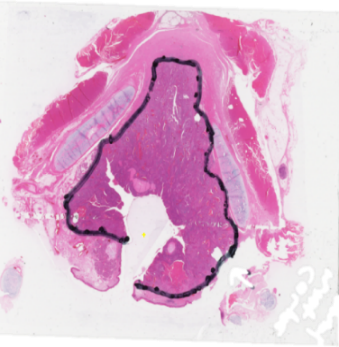

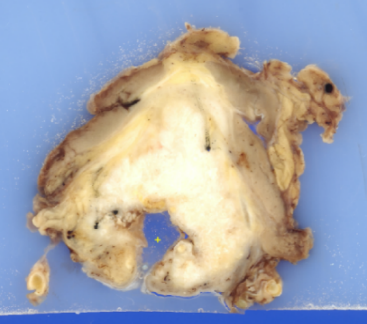


*Figure S1-4: Micro sections were taken from the macro slices with visible tumor and stained for H&E, the four slices that contained the largest tumor diameter were also immunohistochemically stained for Ki-67, CD45 and HIF-1α*

**Tile creation**

The tumor annotation without artifacts on any of the IHC sections was divided into 0.5x0.5 mm^2^ tiles. Tiles located at the border of the tumor or near artifacts were cropped to fit the contour of the tumor annotation (Figure S1-5). This way, tiles do not contain artifacts or information of non-tumorous tissue. If tiles contained less than 50% of usable tumor area after cropping, the tile was removed from analysis to avoid edge artifacts (tile B in Figure S1-5).


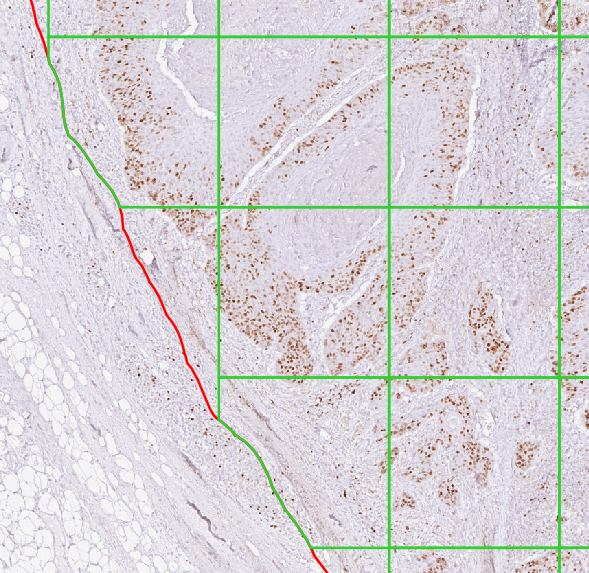


**A**

**B**

*Figure S1-5: 0.5x0.5 mm^2^ tiles (green) are created within the tumor annotation (red). The tiles are cropped to follow the contour of the tumor annotation (tile A). If a cropped tile contains less than 50% of usable tumor area (tile B, black dashed line), this tile is removed from analysis.*

**Heatmap creation**

The positivity of each tile was determined using a threshold. The DAB color signal was isolated using a color deconvolution method^1^.

For each biomarker, a composite image was created in QuPath version 0.3.0^2^ made up of tiles from different tumors. Figure S1-6 A shows this composite image for CD45. The tiles were chosen in such a way that they included areas with high and low biomarker presence, and high and low DAB intensity. A pixel classifier was created based on these composite images. A high resolution (2.02 µm/pixel) was used, and a Gaussian filter with a sigma of 0.5 was added before determining pixel positivity.

A dedicated head and neck pathologist (S.M.W.) visually determined the optimal threshold of the DAB optical density to indicate positive pixels. This threshold was 0.18 for Ki-67, 0.21 for CD45, and 0.20 for HIF-1α staining. Figure S1-6 B shows the result of the pixel classifier, with the positive pixels in red.

The percentage of DAB positivity per tile was used to create heatmaps of all tumor slices.


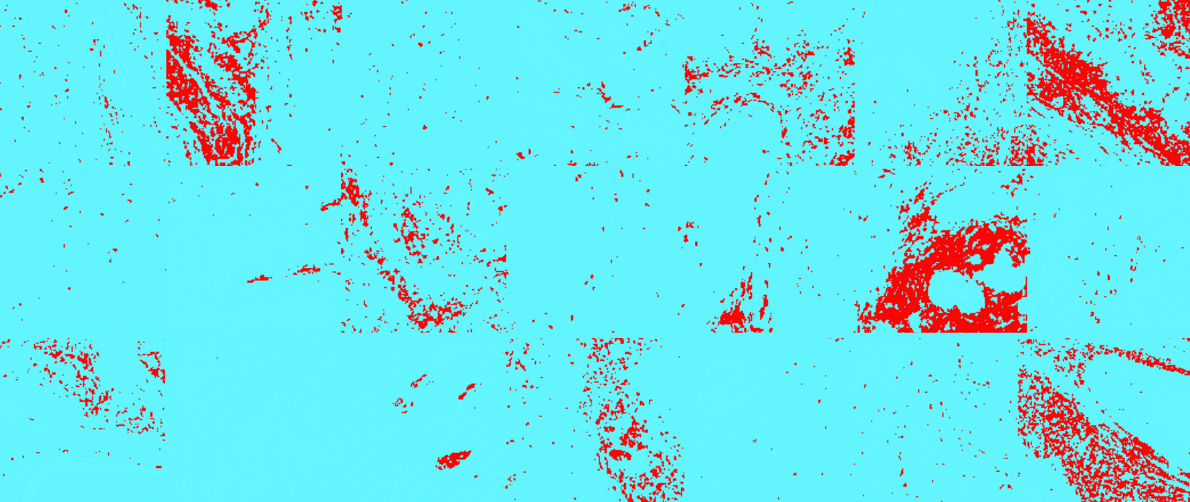

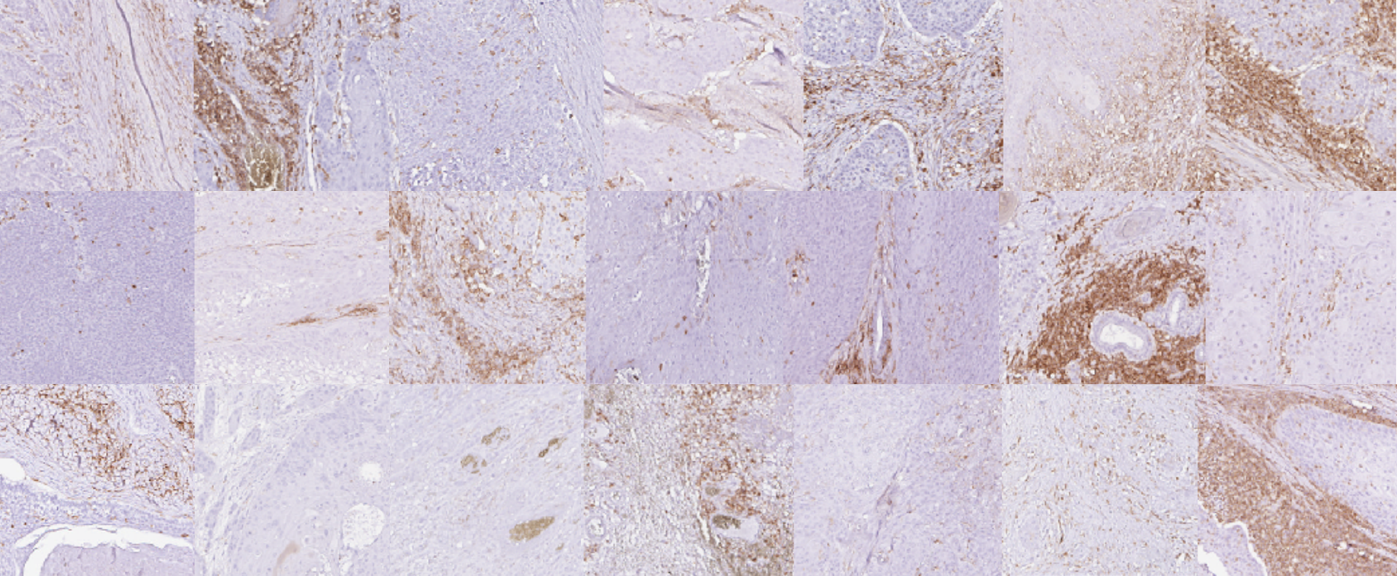


**A**

**B**

*Figure S1-6: Composite image of tiles taken from different CD45-stained tumors (A). Based on this image, the optical density threshold of DAB positivity was determined. The positivity per tile was determined by calculating the amount of positive pixels (red in B).*

**DCE parameter estimation**

The Adiabatic Approximation to the Tissue Homogeneity model^3^ with a fixed transit time of 4 seconds was used for DCE-CT parameter estimation.

In this model, parameter maps are generated for $K^{trans}$, $V_{i}$ (the intravascular space), and $V_{e}$ (the extravascular and extracellular space), Figure S1-7. $K^{trans}$ is the transfer constant which indicates the efflux of contrast agent from the vessels into the tissue. $V_{i}$ is the intravascular space which represents the blood volume in the vessels. $V_{e}$ is the extravascular, extracellular space, which represents the volume in the tissue in which the contrast agent can flow.

The perfusion model also accounts for the reflux of contrast back into the vascular system ($k_{ep}=K^{trans}/V_{e}$). However, since $k_{ep}$ is not a modelled parameter, it was not included in this study.


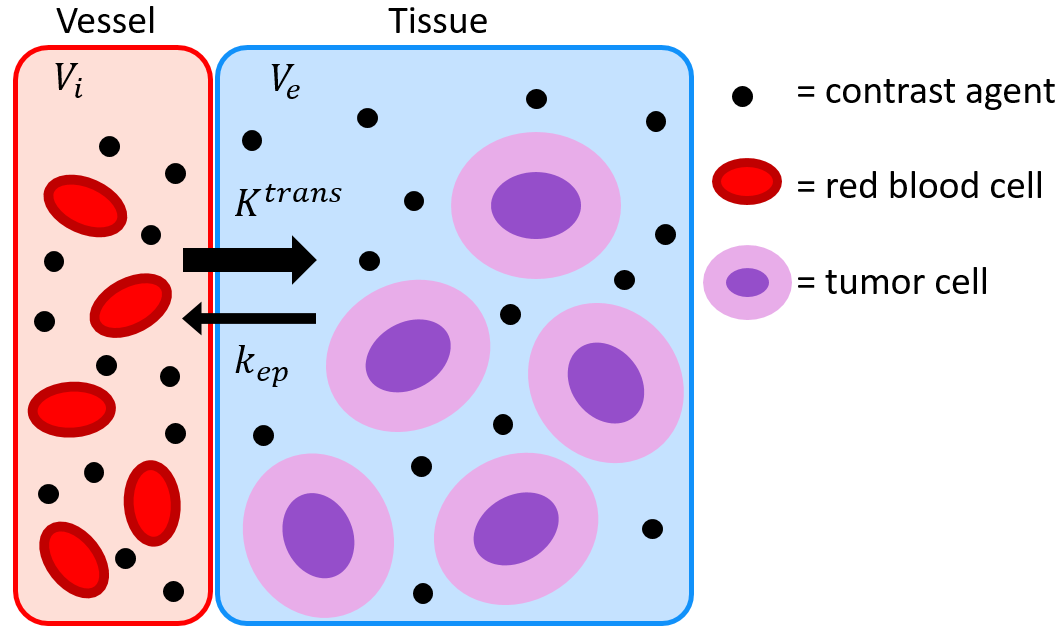


*Figure S1-7: Schematic representation of physiological parameters estimated in the perfusion model.* $V_{i}$ *is the intravascular space,* $V_{e}$ *is the extravascular and extracellular space within the tumor tissue,* $K^{trans}$ *is the transfer constant, the efflux of contrast from vessels into the tissue, and* $k_{ep}$ *is the reflux of contrast from the tissue back into the vessels.*

**Image registration**

Registration of the IHC heatmaps to the H&E slices and the H&E slices to the macro slices was done manually for each individual slice (Figure S1-8). As significant shrinkage of H&E sections has been observed previously^4^, scaling was allowed in this registration step. The IHC heatmaps and the H&E slices were subsequently stacked in a similar manner as the macro slices.

The remaining registrations were done using the Elastix Toolbox^5,6^. The outline of the thyroid and cricoid cartilage was used as a reference to register the 3D macro specimen reconstruction to the ex vivo CT and to register the ex vivo CT to the in vivo CT. Finally, the DCE-CT parameter maps were registered to the in vivo CT.

After registration, the DCE parameter maps were resampled to the IHC heatmaps, using a nearest neighbor interpolation. The DCE parameter maps were cropped to only contain the tumor area and the axial slices from which IHC heatmaps were available.

**IHC heatmap H&E Macro slice**

**DCE parameter map In vivo CT (reference) Ex vivo CT**


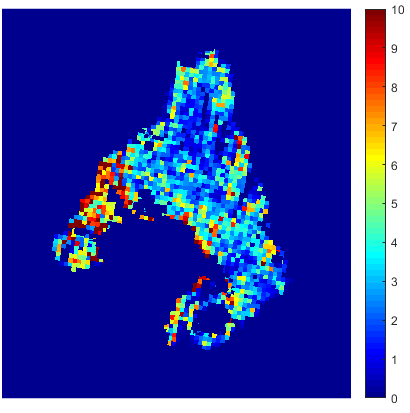

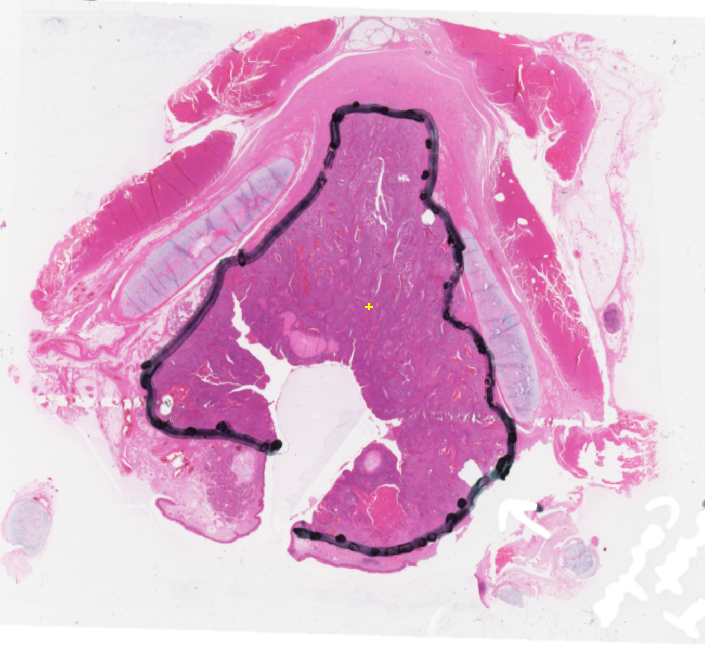

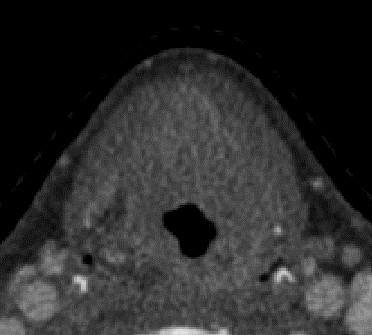

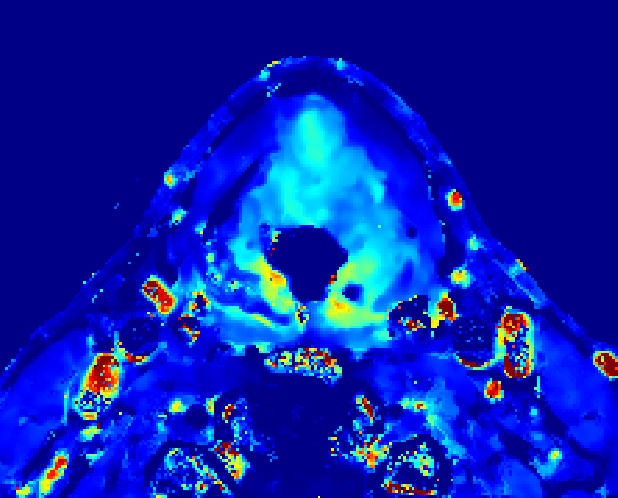

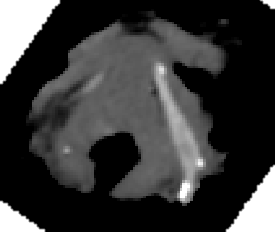

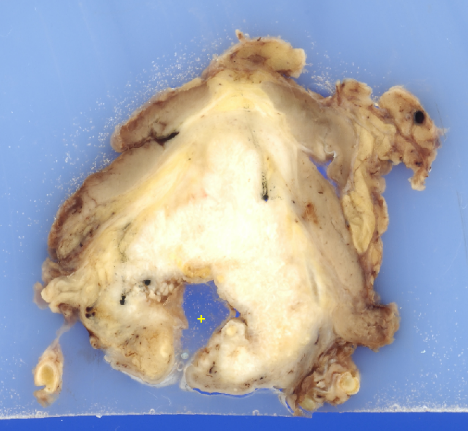


*Figure S1-8: Registration process of immunohistochemistry (IHC) heatmaps to DCE-CT using the in vivo CT as a reference.*

**Heatmap downsampling**

The in-plane resolution was downsampled to create 4x4x3 mm^3^ voxels. If a downsampled voxel only contained 10 or less of the original 0.5x0.5x3 mm^3^ voxels, the voxel was excluded to avoid edge artifacts (Figure S1-9).


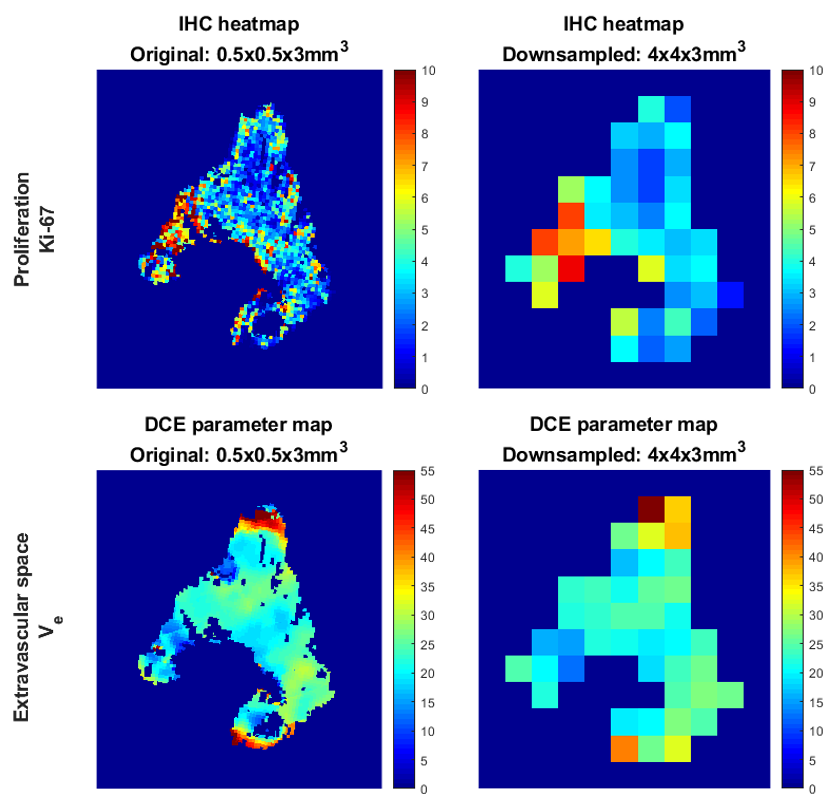

*Figure S1-9: All IHC heatmaps and DCE parameter maps are downsampled to 4x4x3 mm^2^ voxels. The heatmaps show this process for Ki-67 (top, in percentage of positive pixels) and extravascular space (*$V_{e}$*, bottom, in mL/100g) for one tumor slice.*

**References**

1. Ruifrok AC, Johnston DA. Quantification of histochemical staining by color deconvolution. *Anal Quant Cytol Histol*. 2001;23(4):291-299.

2. Bankhead P, Loughrey MB, Fernández JA, et al. QuPath: Open source software for digital pathology image analysis. *Sci Rep*. 2017;7(1):1-7. doi:10.1038/s41598-017-17204-5

3. St. Lawrence KS, Lee TY. An adiabatic approximation to the tissue homogeneity model for water exchange in the brain: II. Experimental validation. *J Cereb Blood Flow Metab*. 1998;18(12):1378-1385. doi:10.1097/00004647-199812000-00012

4. Caldas-Magalhaes J, Kasperts N, Kooij N, et al. Validation of imaging with pathology in laryngeal cancer: Accuracy of the registration methodology. *Int J Radiat Oncol Biol Phys*. 2012;82(2). doi:10.1016/j.ijrobp.2011.05.004

5. Klein S, Staring M, Murphy K, Viergever M a., Pluim J. elastix: A Toolbox for Intensity-Based Medical Image Registration. *IEEE Trans Med Imaging*. 2010;29(1):196-205.

6. Shamonin DP, Bron EE, Lelieveldt BPF, Smits M, Klein S, Staring M. Fast parallel image registration on CPU and GPU for diagnostic classification of Alzheimer’s disease. *Front Neuroinform*. 2014;7(JAN):1-15. doi:10.3389/fninf.2013.00050

Supplementary Material 2 – Parameter cut-offs

Artifacts from the DCE parameter maps were removed from the analysis. To determine the cut-off values for each DCE parameter, histograms were made of all original (not downsampled) 0.5x0.5x3 mm^3^ voxel values from all tumors grouped together (Figure S2-1).

For $TTP$ and $V_{i}$, the cut-off was determined to be 18.5 s and 14.3 mL/100g respectively, as this was two standard deviations ($\sigma$) removed from the mean ($\mu$). For $V_{e}$, $\mu+2\sigma$ was 167.3. However, since $V_{e}$ has a physiological limit of 100 mL/100g, this was chosen as the cut-off for $V_{e}$. Since the $K^{trans}$ parameter maps did not contain outliers, no cut-off point was used for $K^{trans}$. Figure S7 shows the cut-off values as green vertical lines and the maximum values as red lines.

If a tile was zero or exceeded the cut-off for any of these parameters, the tile was completely taken out of the analysis.


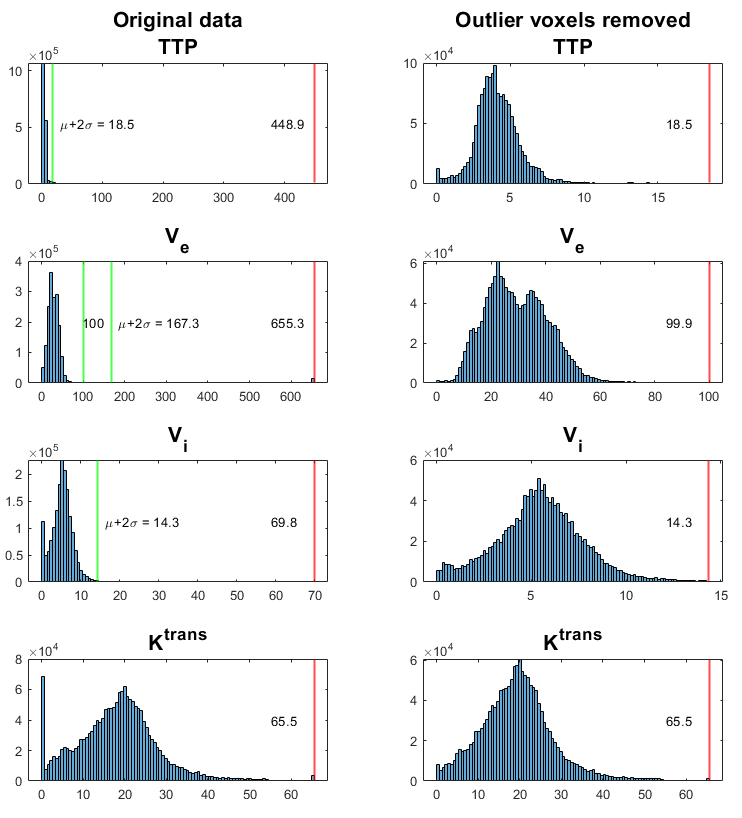


*Figure S2-1: Histograms of DCE parameters of all 0.5x0.5x3 mm^3^ voxels from all tumors. For* $TTP$ *and* $V_{i}$*, values above* $\mu+2\sigma$*, indicated by a green line were removed from analysis. For* $V_{e}$*, the cut-off was placed on the physiological limit of 100 mL/100g. Red indicates the maximum value measured across all voxels.*$TTP$*: time to peak (s),* $V_{e}$*: extravascular and extracellular space (mL/100g),* $V_{i}$*: intravascular space (mL/100g),* $K^{trans}$*: transfer constant (mL/100g/min).*
